# Antibody Repertoire in Paraneoplastic Cerebellar Degeneration and Small Cell Lung Cancer

**DOI:** 10.1371/journal.pone.0060438

**Published:** 2013-03-25

**Authors:** Lidia Sabater, Romana Höftberger, Anna Boronat, Albert Saiz, Josep Dalmau, Francesc Graus

**Affiliations:** 1 Service of Neurology, Hospital Clínic, Universitat de Barcelona and Institut d´Investigació Biomèdica August Pi i Sunyer (IDIBAPS), Barcelona, Spain; 2 Institució Catalana de Recerca i Estudis Avançats (ICREA), IDIBAPS, Hospital Clínic, Barcelona; 3 Department of Neurology, University of Pennsylvania, Philadelphia, Pennsylvania, United States of America; University Hospital of Heidelberg, Germany

## Abstract

The goal of this study is to determine whether patients with paraneoplastic cerebellar degeneration (PCD) and small-cell lung cancer (SCLC) have a specific repertoire of antibodies, if SOX1 antibodies (SOX1-ab) can predict the presence of SCLC, and if antibodies to cell surface antigens occur in this syndrome. Antibody analysis was done using immunohistochemistry on rat brain, immunoblot with recombinant antigens, screening of cDNA expression libraries, and immunolabeling of live neurons in 39 patients with PCD and SCLC. VGCC-ab were measured by RIA, and SOX1-ab, Hu-ab, and ZIC4-ab by immunoblot. Lambert-Eaton myastenic syndrome (LEMS) was present in 10 of 23 patients with electrophysiological studies. At least one antibody was detected in 72% of patients. The individual frequencies were: 49% SOX1-ab, 44% VGCC-ab, 31% Hu-ab, and 13% ZIC4-ab. SOX1-ab occurred in 76% of patients with VGCC-ab and 27% of those without VGCC-ab (p = 0.0036). SOX1-ab were not found in 39 patients with sporadic late-onset cerebellar ataxia, 23 with cerebellar ataxia and glutamic acid decarboxylase antibodies, and 73 with PCD and cancer types other than SCLC (31 without onconeural antibodies, 25 with Yo-ab , 17 with Tr-ab). Five patients (13%) had antibodies against unknown neuronal cell surface antigens but none of them improved with immunotherapy. One serum immunoreacted against the axon initial segment of neurons and another serum against ELKS1, a protein highly expressed in the cerebellum that interacts with the beta4-subunit of the VGCC. In conclusion, 72% of patients with PCD and SCLC had one or more antibodies that indicate the presence of this tumor. In these patients, VGCC-ab and SOX1-ab occur tightly associated. SOX1-ab are predictors of SCLC in ataxia patients with a specificity of 100% and sensitivity of 49%. Unlike limbic encephalitis with SCLC, antibodies to cell surface antigens other than VGCC-ab, are infrequent and do not predict response to treatment.

## Introduction

The Purkinje cell is one of the most common targets of the immune response that some patients with cancer built up against antigens shared by the tumor and the nervous system [1]. The death of Purkinje cells results in a pancerebellar syndrome called paraneoplastic cerebellar degeneration (PCD) [1]. Small-cell lung cancer (SCLC) is one of the most common tumors that associate with PCD [2]. Whereas many patients (>80%) with other paraneoplastic neurological syndromes and SCLC harbor Hu antibodies (Hu-ab), the frequency of Hu-ab in PCD is low (23%) [3]. Approximately, 40% of PCD patients with SCLC have antibodies to voltage-gated calcium channels (VGCC), and some also present clinical or neurophysiological evidence of Lambert-Eaton myasthenic syndrome (LEMS) [3]. Up to 60% of patients with LEMS and SCLC have SOX1-ab, a serologic marker of SCLC [4].

Due to the frequent association of PCD with SCLC, and sometimes with LEMS, we reasoned that determination of SOX1-ab could also be useful to predict whether patients with suspected PCD have an underlying SCLC. Moreover, patients with limbic encephalitis and SCLC but without onconeural antibodies often have antibodies against neuronal surface receptors, if this paradigm also applies for PCD is unknown [5].

In the present study we analyzed the antibody repertoire in a series of patients with PCD and SCLC, focusing on the frequency of SOX1-ab and the presence of novel antibodies to neuronal cell surface antigens.

## Methods

### Patients

We selected from our database, patients with the diagnosis of PCD and SCLC. We specifically excluded patients who presented with ataxia but quickly developed symptoms beyond cerebellar dysfunction. These patients were considered to have paraneoplastic encephalomyelitis, which in contrast to PCD almost always associates with Hu-ab [6]. The neurological disability was evaluated by the modified Rankin scale as described [6], [7]. The clinical information was obtained from forms filled out by the referring neurologists and telephone interviews.

### Standard Protocol Approvals, Registrations, and Patient Consents

Serum and CSF samples used in the study are deposited in the collection of biological samples named "neuroinmunología" registered in the biobank of Institut d' Investigació Biomèdica August Pi i Sunyer (IDIBAPS), Barcelona, Spain. Considering that many patients were dead at the time the study was performed and the study is completely anonymous so no sample can be identified to a particular patient, it was accepted to waive the specific written informed consent from the patients or next of kin by the Comitè Etic d'investigació Clínica (CEIC) of Hospital Clínic. Animal handling procedures were approved by the Local Ethics Committee (99/1 University of Barcelona) and the Generalitat de Catalunya (1094/99), in accordance with the Directive 86/609/EU of the European Commission. The study as explained was approved by the CEIC of Hospital Clínic.

### Detection of anti-neuronal antibodies

Serum and CSF, when available, were evaluated for the presence of onconeural (Hu, Yo, Ri, CV2, amphiphysin, Ma2, Tr), anti-neuropil (NMDAR, AMPAR, GABA_B_R, CASPR2, LGI1, mGluR1 and mGluR5) or possible new antibodies by immunohistochemistry on frozen sections of paraformaldehyde-perfused or post-fixed rat cerebellum as reported [8]. Onconeural antibody positivity was confirmed by a commercial immunoblot (Ravo Diagnostika GmbH, Freiburg, Germany) and anti- neuropil antibodies by a cell based assay using HEK293 cells transfected with the appropriate plasmids, as previously described [9]. Antibodies against the subunit β4 and γ2 of VGCC were detected by immunofluorescence on HEK293 cells transfected with the plasmids (MC201619 and SC312948; Origene, Rockville, Maryland, USA). Antibodies to P/Q type VGCC were measured by a commercial radioimmunoassay (DLD Diagnostica GMBH, Hamburg, Germany). SOX1-ab and ZIC4-ab were determined using nitrocellulose filters with mixed phage plaques (50% of plaques from positive clones and 50% from irrelevant clones). Filters were cut into pieces, each one incubated with different patient’s sera (dilution 1∶1000) and developed by an avidin-biotin immunoperoxidase technique as described [4].

To confirm the specificity of SOX1-ab in the diagnosis of PCD associated with SCLC, SOX1-ab were also analyzed in the serum of 31 patients with PCD and different cancer types, other than SCLC, that did not harbor onconeural antibodies that could confirm the diagnosis of PCD, 25 with PCD, breast or ovarian cancer, and Yo-ab, 17 with PCD, Hodgkin disease, and Tr-ab, 39 patients with sporadic late-onset cerebellar ataxia, and 23 with cerebellar ataxia and glutamic acid decarboxylase antibodies.

### Primary cell culture and in vivo immunocytochemistry

Hippocampal neurons were obtained from E18 Wistar rat embryos. Cells were enzymatically and mechanically disrupted and resuspended in Neurobasal medium supplemented with B27 (Invitrogen, Carlsbad, CA, USA) [9]. Granular cell cultures were prepared from dissected cerebella of 8-day-old Wistar rats and processed equally as hippocampal neurons but using Neurobasal-A medium containing 25 mM of KCl and supplemented with B27 [10]. Cells were plated in poly-L-lysine pre-coated P24 plates and 10 µM cytosine -D-arabinofuranoside (Sigma-Aldrich, St. Louis, MO, USA) was added to the cultures 20 h after plating, to prevent proliferation of non neuronal cells.

In the immunocytochemistry experiments, samples (1∶5 CSF dilution and 1∶200 serum dilution) were incubated on live neurons for 1 hour, then fixed with 4% paraformaldehyde and permeabilized with 0.3% Triton TX-100. Staining with anti-MAP2 antibody was performed in order to confirm the neuronal specificity of the positive cells. Appropriate fluorescent secondary antibodies were applied and the coverslips were mounted with Vectashield with DAPI mounting media (Vector Laboratories, Burlingame, CA, USA) and visualized with an Axio Imager M2 ZEISS microscope.

### Screening of cDNA expression libraries

Uni-ZAP XR libraries from human cerebellum and human fetal brain (Stratagene, La Jolla, CA) were immunoscreened with 10 PCD SCLC sera (4 patients with SOX1-ab and VGCC-ab, 4 seronegative, 1 with VGCC-ab and 1 with SOX1-ab), using techniques previously reported [4]. Briefly, immunoscreening of the cDNA library with sera (dilution 1∶1000) was performed to reach isolated positive plaques. Phage clones were subcloned in pBluescript plasmid using the in vivo excision phage rescue protocol (Stratagene, La Jolla, CA). Plasmid DNA was purified with the QIAprep Spin Miniprep Kit (Qiagen, Santa Clarita, CA), and sequenced with the DNA sequencer ABI 377 (Applied Biosystems, Foster City, CA) using the Big Dye terminator ready mix (Applied Biosystems). Positive clones were sequenced on both strands. The human genome BLAST program (NCBI, NIH, Bethesda, MD) was used to search for homologies.

### Statistical Analysis

The correlation between SOX1-ab and VGCC-ab were analyzed in a contingency table and the two-tailed Fisher exact test was applied.

## Results

### Patients

We identified 39 patients with PCD and SCLC. Median age of the patients was 64 years (range: 47 to 85 years) and 34 were male. PCD antedated the diagnosis of SCLC in 36 patients with a median time of 2 months (range: 0.5 to 42 months). At the time of PCD diagnosis 10 patients had LEMS and 13 did not (confirmed by at least normal compound muscle action potentials in electromyography (EMG) studies [11]). The rest of the 16 patients did not refer symptoms of LEMS but no EMG studies were done. Twenty seven patients received at least one full course (intravenous immunoglobulins or steroids, or both) of immunotherapy (6 patients), chemotherapy (14) or both (7). No improvement or stabilization with moderate-severe cerebellar dysfunction (Rankin score > 3) occurred in 13 of the 21 patients (62%) who had adequate follow-up. Three patients improved and 5 remained stable with a Rankin score of 3 or less.

### VGCC and onconeural antibodies: association with SOX1 antibodies

**Figure 1:**
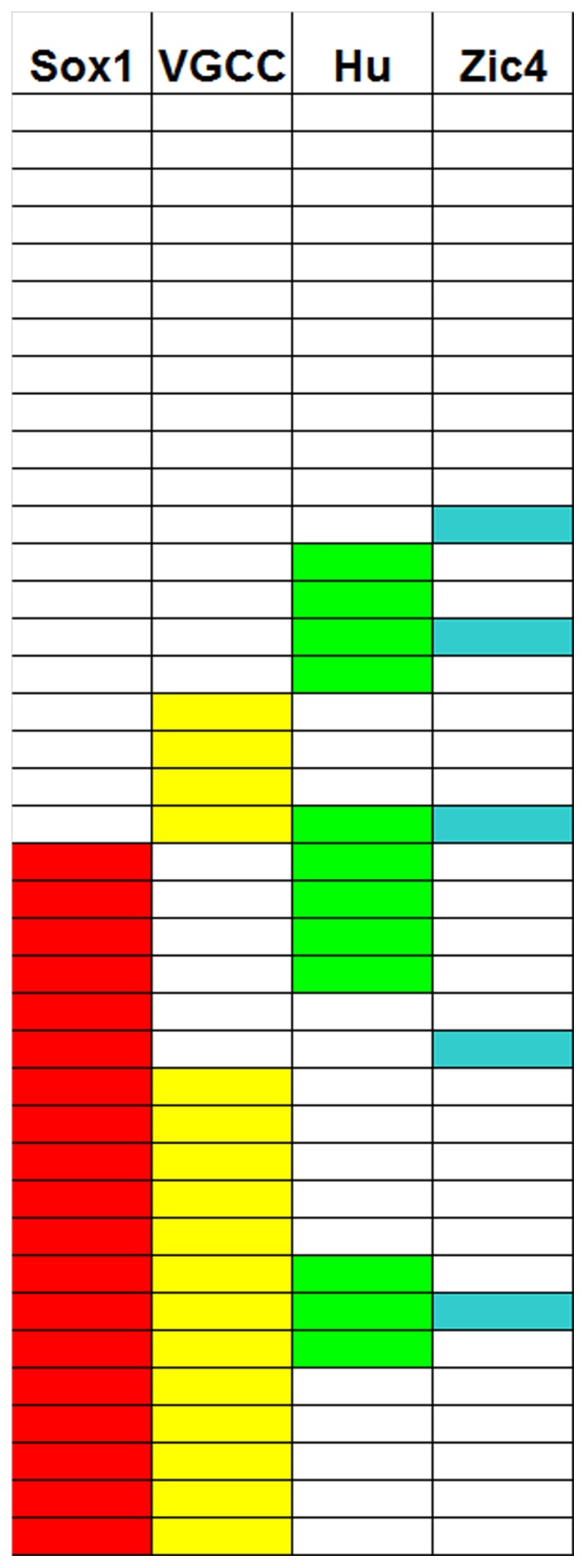
Graphic representation of the SOX1, VGCC, Hu, and ZIC4 antibodies in PCD associated with SCLC. Distribution of SOX1-, VGCC-, Hu-, and ZIC4-ab in our patient cohort. Each row represents a patient and when positive the cell is filled in color.

**Figure 2:**
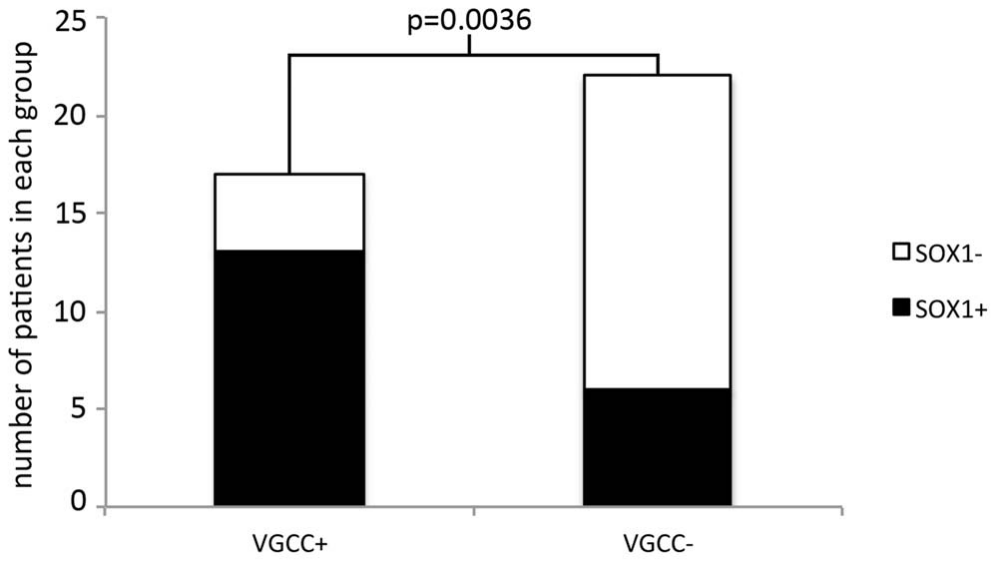
Association of VGCC and SOX1 antibodies. Bar graph representation of the correlation between SOX1-ab and VGCC-ab. (Fisher exact test p = 0.0036).

Seventy-two percent of patients had at least one of the indicated antibodies. The individual frequencies of the antibodies were: 49% SOX1-ab, 44% VGCC-ab, 31% Hu-ab, and 13% ZIC4-ab. Twenty eight sera presented at least one antibody, and 11 were negative (Figure 1). There was a clear correlation between SOX1-ab and VGCC-ab. SOX1-ab were found in 76.5% of patients with VGCC-ab but in only 27.3% of those without VGCC-ab (Fisher exact test p  =  0.0036) (Figure 2). Positive VGCC-ab were found in 9 (90%) of the 10 patients with LEMS and in 7 (54%) of the 13 without clinical or EMG evidence of LEMS. Similarly, SOX1-ab were present in 80% of patients with LEMS and 38% of those without LEMS. No associations were observed between VGCC-ab or SOX1-ab and Hu-ab. None of the patients were positive for onconeural antibodies, other than Hu, or presented well characterized anti-neuropil antibodies. SOX1-ab were not detected in the control series of 73 patients with PCD and different types of cancers other than SCLC, 32 patients with sporadic late-onset cerebellar ataxia, and 23 with cerebellar ataxia and glutamic acid decarboxylase antibodies.

### Identification of new antibodies

**Figure 3:**
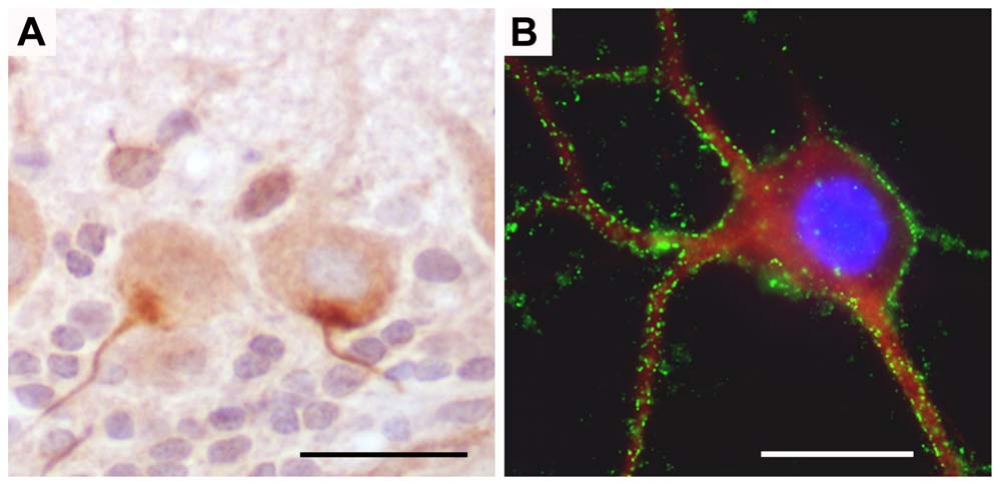
Identification of new antibodies. A. Antibodies against hillock of the neurons of rat cerebellum. Immunohistochemistry on rat sagittal cerebellum sections. Note the strong atypical reactivity of a PCD SCLC serum against the hillock axon of the neurons, mainly in Purkinje cells. Nuclei are counterstained with hematoxilin. B. Immunofluorescence on live hippocampal neurons. Hippocampal neurons incubated with CSF 1/5 diluted of a PCD SCLC patient. Reactivity of the patient against the membrane of the neuron is seen in green and MAP2 staining in red to ensure the neuronal lineage. Nuclei are seen blue with DAPI. (objective 100x oil)

One serum with SOX1-ab also showed robust immunoreactivity against the axon initial segment of neurons in routine immunohistochemical studies (Figure 3A). Screening of a human cerebellum cDNA expression library with this serum failed to identify the putative antigen. Another new antibody was identified during the screening of cDNA libraries with one serum with VGCC-ab. The target protein, called ELKS1, is highly abundant in the cerebellum. We performed a specific phage screening assay for the detection of ELKS1 but we could not find other positive sera. PCD sera also did not show immunoreactivity with HEK293 cells transfected with the β4 or γ2 auxiliary subunits of VGCC. Five patients (12.8%) had antibodies against cell surface antigens expressed in live, non-permeabilized hippocampal neurons (Figure 3B), but only 1 of them reacted with cerebellar granular neurons.

## Discussion

In the current study we wanted to determine if patients with PCD and SCLC had antibodies other than VGCC-ab that could explain the cerebellar ataxia or at least to help in the diagnosis of PCD. The isolated determination of VGCC-ab in a patient with cerebellar ataxia with or without LEMS almost always confirms a paraneoplastic origin because the clinical association of cerebellar ataxia and LEMS very rarely occur in absence of cancer [12], [13]. In addition, if the cerebellar ataxia is paraneoplastic and the patient has VGCC-ab the underlying tumor is always a SCLC [14].

To overcome this limitation, we analyzed the presence of SOX1-ab, a serological marker of SCLC [15]. In our series of PCD and SCLC we found that SOX1-ab were present in almost 50% of these patients. In contrast, SOX1-ab were not found in serum of patients with PCD and other tumor types with or without onconeural antibodies and in patients with cerebellar ataxia without cancer. Our data indicate that SOX1-ab are good predictors of an underlying SCLC in patients with cerebellar ataxia. The frequency of SOX1-ab was even higher (76.5%) in PCD patients with SCLC who also had VGCC-ab. This frequency is similar to that reported in patients with LEMS and SCLC (67%) [16]. Taken together, these data suggest that in patients with PCD and SCLC, the co-occurrence of VGCC-ab and SOX1-ab is strongly associated even though 41% of PCD patients with VGCC-ab of the current series did not have LEMS.

Previous studies showed that all patients with LEMS and SCLC who had Hu-ab also had SOX1-ab [16]. Therefore, additional testing for Hu-ab did not improve the diagnosis of paraneoplastic LEMS [16]. However, the finding of the current study has different implications. Whereas SOX1-ab, occurred in 48.7% of patients with PCD, an additional 20% of patients had Hu-ab or VGCC-ab. Overall, combining the testing of all 3 antibodies (all commercially available) will identify almost 70% of patients with PCD and SCLC. A few patients may have other onconeural antibodies like ZIC4, CV2 or amphiphysin and all but ZIC4 are routinely screened with the same kits commonly used to detect Hu-ab.

We did not find other potentially useful antibodies in this series. The serum of one patient showed reactivity with the axon initial segment of large neurons. This antibody was previously reported in a patient with encephalomyelitis and SCLC who developed seizures and severe gait ataxia [17]. We previously identified this immunoreactivity in a patient with SCLC without neurological symptoms suggesting the antibody may be a marker of the underlying SCLC (unpublished). Another patient had an antibody against ELKS1, a protein predominantly expressed in Purkinje cells [18]. Although ELKS1 co-immunoprecipitates with the β4 VGCC subunit, we could not identify antibodies reacting directly with β4 or γ2, which are VGCC subunits that are highly expressed in the cerebellum [18], [19]. Our study does not rule out the possibility that the sera of these patients could have antibodies against other VGCC subunits or interacting proteins. For example, sera from patients with non-paraneoplastic LEMS react more frequently with the domain IV of the alpha1A of VGCC compared with patients with LEMS and SCLC [20].

The recent discovery of antibodies against neuronal surface antigens has changed the concept that antibodies in CNS paraneoplastic syndromes are only surrogate markers of the underlying tumor. While in patients with limbic encephalitis and SCLC approximately 50% have Hu-ab and the other 50% have antibodies against synaptic proteins (mainly GABA_B_ receptor) [5], our study shows that in patients with PCD and SCLC the occurrence of antibodies to cell surface proteins other than VGCC-ab, is rare.

The practical implication of our study is that in patients with cerebellar ataxia, SOX1-ab are good predictors of an associated SCLC with a specificity of 100% and a sensitivity of 49%. In addition, the combination of testing for SOX1-, VGCC-, and Hu-ab identifies almost 70% of patients with PCD and SCLC. Given that PCD usually develops before the diagnosis of the tumor, this repertoire of antibodies is useful to establish that the cerebellar syndrome is paraneoplastic, the underlying tumor is SCLC, and the chance of neurological recovery is low.
